# The molecular mechanisms of calcium signalling in *Chlamydomonas* flagella

**DOI:** 10.1186/2046-2530-1-S1-P19

**Published:** 2012-11-16

**Authors:** PW Collingridge, G Wheeler, C Brownlee

**Affiliations:** 1 The Marine Biological Association of the United Kingdom, UK

## 

The motile green alga, *Chlamydomonas*, has long been used as a model system to understand flagella structure and function. In addition to the well characterised roles of cilia and flagella in motility, cell biologists are becoming increasingly aware of their importance as cellular sensors. Ca2+-dependent signalling mechanisms are associated with many flagellar processes, although in many cases the underlying mechanisms remain unclear. Whilst *Chlamydomonas* possesses many genetic and biochemical advantages for the study of flagella, imaging Ca2+ in this alga has proven to be problematic. We have developed techniques to introduce Ca2+ responsive fluorescent dyes into *Chlamydomonas* cells via biolistics, which enables us to routinely and robustly image Ca2+ in both the cytosol and in the flagella. To visualise Ca2+ elevations within the flagella, we have developed imaging techniques using Total Internal Reflectance Fluorescence (TIRF) microscopy. This approach allows us to specifically image fluorescence from the flagella at high spatial and temporal resolution in the absence of interfering fluorescence from the cell body. We have found that *Chlamydomonas* flagella exhibit a range of dynamic Ca2+ elevations in response to different stimuli. Each flagellum demonstrates the ability to generate Ca2+ elevations independently from each other, suggesting a level of control in *Chlamydomonas* flagella signalling processes not previously demonstrated. We are currently using RNAi approaches to assess the contribution of different flagella-localised ion channels to these Ca2+ signalling processes.

